# Golden Tomato Juice Enhances Hepatic PPAR-α Expression, Mitigates Metabolic Dysfunctions and Influences Redox Balance in a High-Fat-Diet Rat Model

**DOI:** 10.3390/antiox13111324

**Published:** 2024-10-30

**Authors:** Danila Di Majo, Nicolò Ricciardi, Alessandra Moncada, Mario Allegra, Monica Frinchi, Valentina Di Liberto, Rosa Pitonzo, Francesca Rappa, Filippo Saiano, Filippo Vetrano, Alessandro Miceli, Giuseppe Giglia, Giuseppe Ferraro, Pierangelo Sardo, Giuditta Gambino

**Affiliations:** 1 Department of Biomedicine, Neuroscience and Advanced Diagnostics (BIND), University of Palermo, 90127 Palermo, Italy; nicolo.ricciardi@unipa.it (N.R.); monica.frinchi@unipa.it (M.F.); valentina.diliberto@unipa.it (V.D.L.); francesca.rappa@unipa.it (F.R.); giuseppe.giglia@unipa.it (G.G.); giuseppe.ferraro@unipa.it (G.F.); pierangelo.sardo@unipa.it (P.S.); giuditta.gambino@unipa.it (G.G.); 2 Postgraduate School of Nutrition and Food Science, University of Palermo, 90100 Palermo, Italy; mario.allegra@unipa.it; 3 Department of Agricultural, Food and Forest Sciences (SAAF), University of Palermo, 90128 Palermo, Italy; alessandra.moncada@unipa.it (A.M.); filippo.saiano@unipa.it (F.S.); filippo.vetrano@unipa.it (F.V.); alessandro.miceli@unipa.it (A.M.); 4 Department of Biological, Chemical and Pharmaceutical Sciences and Technologies (STEBICEF), University of Palermo, Viale delle Scienze, 90128 Palermo, Italy; 5 ATeN (Advanced Technologies Network) Center, 90128 Palermo, Italy; rosa.pitonzo@unipa.it; 6 Euro Mediterranean Institute of Science and Technology (IEMEST), 90139 Palermo, Italy

**Keywords:** oxylipin, metabolic syndrome, PPAR-α, leptin, oxidative stress, functional food

## Abstract

Golden tomato (GT), harvested at the veraison stage, has gained attention due to its rich content of bioactive compounds and potential health benefits. Previous studies have highlighted GT’s antioxidant properties and its positive effects on metabolic syndrome (MetS), a condition characterized by obesity, dyslipidemia, and oxidative stress. This study investigates for the first time a derivative from GT, i.e., the juice (GTJ), which could be a potential candidate for development as a functional food. We first characterized GT juice, identifying 9-oxo-10(E),12(E)-octadecadienoic (9-oxo-10(E),12(E)-ODA) fatty acid, a known peroxisome proliferator-activated receptor alpha (PPAR-α) agonist, using High-Performance Liquid Chromatography (HPLC)–mass spectrometry. Then, using a high-fat-diet (HFD) rat model, we assessed the impact of daily GT juice supplementation in addressing MetS. We outlined that GTJ improved body weight and leptin-mediated food intake. Moreover, it ameliorated glucose tolerance, lipid profile, systemic redox homeostasis, hepatic oxidative stress, and steatosis in HFD rats. Furthermore, GT juice enhances the hepatic transcription of PPAR-α, thus putatively promoting fatty acid oxidation and lipid metabolism. These findings suggest that GT juice mitigates lipidic accumulation and putatively halters oxidative species at the hepatic level through PPAR-α activation. Our study underscores the protective effects of GT juice against MetS, highlighting its future potential as a nutraceutical for improving dysmetabolism and associated alterations.

## 1. Introduction

Metabolic syndrome (MetS) is a widespread cluster of risk factors associated with obesity, cardiovascular disease, and disrupted oxidative balance conditions that can significantly exacerbate existing comorbidities [1,2,3,4,5]. The diagnosis of MetS occurs when three or more of the following clinical markers are present: hypertension, atherogenic dyslipidemia, increased visceral adiposity, and hyperglycemia or insulin resistance [6,7]. Over the past decade, research has suggested the potential of PPAR-α agonists in addressing MetS-related risk factors and associated inflammation [8]. Increasingly, the lipo-inflammation characteristic of MetS is considered a key complication driving health deterioration, with PPAR-α emerging as a possible link between nutrition, metabolic organs, and the immune system [8] In animal models, MetS can be induced by employing a special diet regimen, i.e., a high-fat diet (HFD), that reproduces the complete clinical manifestations in terms of increased body weight, reduced food intake, glucose tolerance, and dyslipidemia [9,10,11]. Our previous research revealed that specific systemic biomarkers of redox homeostasis are robustly linked to the development of metabolic dysfunctions, strengthening the impact of oxidative-based alterations in MetS [11].

A growing interest has arisen in the use of nutraceuticals contained in foods that can counteract MetS [12,13]. In this context, tomato fruit stands out as a food of the Mediterranean diet that represents an excellent source of nutrients and bioactive compounds, the concentrations of which are related to the prevention of chronic degenerative diseases such as cardiovascular disorders, cancer, and neurodegenerative diseases [14,15]. The European Food Safety Authority (EFSA) has already approved a health claim for the water-soluble tomato concentrate extract registered as Fruitflow^®^: ‘Helps maintain normal platelet aggregation, which contributes to healthy blood flow’ [15]. In addition, other studies have attributed beneficial effects to fresh tomatoes and their derivatives [15]. Among them, Golden Tomato (GT) is produced from a mixture of Brigate tomatoes harvested at the same time and in the same area, with different degrees of ripeness and coloring with respect to red tomatoes (RT), hence the name “golden”. The characterization and the nutraceutical properties of the fruits utilized for the production of GT have been published by our group [14]. Indeed, we highlighted the beneficial effects of GT on systemic redox balance, lipid and glycaemic homeostasis, non-alcoholic fatty liver disease (NAFLD), and body weight control in MetS [14,16]. Namely, genetic studies in a rodent model have shown preferential effects of GT compared with RT on the expression of genes in the liver, such as hepatocyte nuclear factor 4 alpha (HNF4α) and glycerol kinase (GK), which are involved in lipid metabolism, and the leptin receptor (Lepr) gene, which is also involved in the inflammatory response, revealing the potential protective role of golden tomato supplementation in NAFLD [16]. Furthermore, we also studied the central protective effects due to the administration of the juice from Golden Tomato in an HFD rat model, ameliorating cognitive dysfunction and modulating neuroinflammatory signaling associated with MetS, acting through PI3K/Akt and MAPK/ERK pathways [17].

As for peroxisome proliferator-activated receptor alpha (PPAR-α), it has been characterized as a key moderator regulating metabolic adaptation to fatty acid increase. Its activation results in hepatic fatty acid oxidation and also stimulates cellular uptake of fatty acids by increasing the expression of fatty acid transport protein (FATP) and fatty acid translocase (FAT) [18]. PPAR-α is responsible for lipid homeostasis in the liver through the regulation of the catabolic and synthetic pathways, thereby reducing the cytotoxic effects of free fatty acids. PPAR-α is highly expressed in the liver, and activation of this receptor promotes the expression of cytochrome P4504A (CYP4A), a subclass of cytochrome P450 that catalyzes the ω-hydroxylation of fatty acids [19], which is useful in reducing triglyceride (TG) synthesis. Endogenous and exogenous PPAR-α agonists are used in the treatment of dyslipidemia and type II diabetes mellitus. In the last decade, it has been shown that PPAR-α agonists can be used therapeutically to treat risk factors associated with metabolic syndrome and inflammation. Relevantly, it was recognized in the literature that human and rodent livers have comparable levels of PPAR-α that vary over the course of the day [20]. Importantly, tomato and its derivatives contain 9- and 13-oxo-octadecadienoic acids, which act as potent activators of PPAR-α, strongly reducing the level of triglycerides in primary rat hepatocytes, and are classified as oxylipin [16,21]. In plants, oxylipin comes from the oxidation of polyunsaturated fatty acids, such as linoleic acid, and acts as a second messenger in interorganismic signaling and as a bactericide [22].

In light of this, the present study aims to investigate the potential of Golden Tomato Juice (GTJ) as a functional food in mitigating the harmful effects of MetS. This research focuses on two main objectives. Firstly, we point to the characterization and quantification of GTJ bioactive compounds, focusing on the presence of the 9-oxo-10(E),12(E)-octadecadienoic fatty acid (9-oxo-10(E),12(E)-ODA), a known PPAR-α agonist via High-Performance Liquid Chromatography (HPLC)–mass spectrometry. Once provided with a fingerprint of nutraceuticals in the GTJ, our subsequent aim was to assess the effects of oral daily administration of GTJ on a rat model of MetS induced by a hyperlipidemic diet. In detail, we explored the influence of GTJ on key parameters obtained from lipid and glycemic homeostasis, body weight control, systemic and liver redox homeostasis, and hepatic steatosis. Additionally, this study will investigate whether GTJ is able to modulate the PPAR-α expression at the transcriptional level in the liver, seeking a molecular basis for its potential health benefits. By addressing these goals, this study aims to demonstrate the protective effects of Golden Tomato Juice on metabolic syndrome and to explore its potential as a dietary intervention for improving metabolic health.

## 2. Materials and Methods

### 2.1. Tomato Seedling Cultivation and Morphological Characteristics

The seedlings of tomato (Solanum lycopersicon cv. Brigade) were transplanted on 12 May 2021 at a density of 8300 plants/ha (0.60 × 2 m). The crop was irrigated and cultivated according to organic farming practices. Some morphological parameters of plants and fruits were observed during plant growth and fruit ripening: plant growth rate, beginning of flowering and fruiting, number of fruits of the first truss, yield at physiological maturity, and different ripening stages.

### 2.2. Process of Turning Golden Tomatoes into Juice

Soon after harvesting, the fruits were transferred to the “Cooperativa Rinascita” (Sclafani Bagni, PA, Italy) for tomato processing. The protocol for the preparation of the GTJ involved the following phases: washing fruits in a bubbling tank by insufflation of air to detach any residues; sorting on a conveyor belt to eliminate unsuitable fruit and foreign bodies; chopping and transferring the product to a heat exchanger (hot break) at 85 °C for enzymatic inactivation; and extraction and refining through strainers–refiners to separate the juice from the cellulose parts (epicarp and seeds) by centrifugal sieving (0.6 mm sieve) and tube pasteurization treatment at 95 °C for 90 s. The refined juice was transferred to the volumetric glass jar filler and subsequently to the pasteurization tunnel (at 92 °C for 15 min), followed by cooling. 

#### 2.2.1. Chemical and Nutritional Properties of Golden Tomato Juice

The GTJ was characterized both chemically by assessing the titratable acidity, pH, Brix degree, dry matter, total nitrogen, and total polyphenols, as well as nutritionally by analyzing the macronutrients (proteins, fats, and carbohydrates) and micronutrients (mineral salts and some vitamins). In addition, an investigation of certain organic acids (citric, malic, tartaric, oxalic) and amino acids such as glutamic acid, which regulate the acid-base balance of the juice, was carried out.

Analyses were performed according to the official methods of analysis of the Association of Official Analytical Chemists (AOAC) [23]. The equations used to calculate the proximal analysis results are given in Table 1. The analysis of organic acids was performed by HPLC, which provides good separation and quantification of the products according to Cadavid’s method [24].

- Energy calculation

The energy content of the juice sample was calculated according to Atwaters’ protocol. Energy content was calculated by the following equation [25]:

Energy content = (% protein × 4) + (% carbohydrates × 4) + (% fat × 9)

#### 2.2.2. Analysis of Total Polyphenols by Folin Ciocalteu Assay in GTJ

The total polyphenolic content was evaluated using the Folin Ciocalteu assay with the use of a specific commercial kit and the Free Carpe Diem device (FREE^®^ Carpe Diem; Diacron International, Grosseto, Italy), as detailed in the previous paper [14].

#### 2.2.3. Identification and Quantification of 9-Oxo-10(E),12(E)-Octadecadienoic Acid in GTJ by HPLC System

The analysis of 9-oxo-10(E),12(E)-ODA in freeze-dried GTJ involved an initial extraction step followed by an identification and quantification step using HPLC.

- Sample preparation

It was carried out on 20 mg of freeze-dried tomato juice. The freeze-dried lyophilizate was sonicated for 10 min at 25 Hz, cold, after the addition of 2 mL EtOH, using an ultrasonic homogenizer (Model 150V/T, Biologics Inc., Manassas, VA, USA). The resulting solution was centrifuged using a Centra-MP4 centrifuge (Cat.n° 2437, IEC) at 15.000 rpm, T = 4 °C, for 10 min. The supernatant was collected, and the residue was subjected to further extraction under the same conditions. The supernatants of the two extractions were combined and filtered through 0.2 µm pore PVDF filters. The filtrate was used for the fatty acid identification and quantification steps. The extract to be analyzed in HPLC has a final concentration of 20 mg in 4 mL of methanol.

- Identification and quantification of 9-oxo-10(E),12(E)-ODA by HPLC

An Ultra-High-Performance Liquid Chromatography (UHPLC-Q) Exactive Orbitrap–HRMS (Hybrid Quadrupole-Orbitrap Mass Spectrometers) system (Thermo Fisher Scientific™, Bremen, Germany) composed of a Dionex Ultimate 3000 liquid chromatograph coupled to a Q Exactive™ Plus Hybrid Quadrupole-Orbitrap™ Mass Spectrometer equipped with a heated electrospray ionization (HESI) ion source was used to analyze tomato extract samples. Chromatographic separation was achieved on Kinetex F5 (100 × 2.0 mm, 1.7 μm) equipped with a precolumn; the column was set to 30 °C. Mobile phases used were water (mobile phase A) and methanol (mobile phase B). A gradient method at 200 μL/min flow rate was applied as follows: start at 60% B, stay for 2 min; increase to 100% B over 8 min, hold for 7 min; then decrease to 60% B over 2 min; and maintain constant for 3 min, for a total run time of 20 min. Injection volume was 1 μL. Full mass and targeted SIM (t-SIM) scan methods were applied. The Orbitrap parameters were set as follows: negative (−) ESI full scan mode and t-SIM, sheath gas flow rate 30 AU, discharge voltage 2.5 kV, capillary temperature 280 °C, resolution 35,000 FWHM, AGC target 5 × 10^6^, maximum injection time 200 ms, and scan range 100–500 m/z. Calibration curve was constructed at five calibration levels for the standard 9-oxo-10(E),12(E) octadecadienoic acid in the range from 0.1 to 1 µg/mL.

### 2.3. Animals

**Figure 1:**
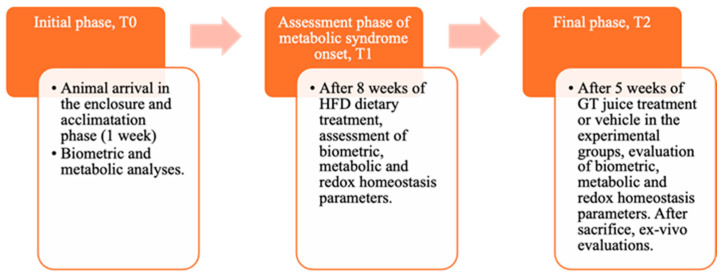
Graphic representation of the main phases of the experiment, indicating the biochemical and anthropometric analysis carried out.

Male Wistar rats (4-week-old), weighing 240–260 g, were obtained from. Envigo RMS B.V. (The Netherlands) They were housed in pairs per cage and kept on a 12 h light/dark cycle (8:00–20:00 h) at a stable temperature (22–24 °C) and humidity (50 ± 10%). During the acclimation period, animals were initially fed with a standard chow diet providing 3.94 kcal/g and then separated into two homogenous groups with balanced weights. These groups were either fed with standard laboratory food (NPD: normal pellet diet, code PF1609, certificate EN 4RF25, Mucedola, Milan, Italy) or with high-fat-diet (HFD) food, where 60% of energy came from fats (code PF4215-PELLET, Mucedola, Milan, Italy) to induce MetS, as determined by established criteria in the literature [10,11]. The detailed composition of the diet is provided in our previous study [11]. All rats had free access to food and water. Before starting the special diet, all animals were weighed. The experiment consisted of three stages, To, T1, and T2, defined according to procedures detailed in the graphical representation in the following Figure 1.

Animal care and handling were in compliance with the European Communities Council Directive (2010/63/EU). The experimental protocols were approved by the animal welfare committee of the University of Palermo, authorized by the Ministry of Health (Rome, Italy; Authorization Number 14/2022-PR), and conducted following the ARRIVE guidelines.

#### 2.3.1. Experimental Groups

In T0, animals were initially divided into NPD or HFD on the basis of the diet provided and maintained for 8 weeks until MetS induction. In T1, after confirming the induction of MetS following detailed procedures [11], animals were divided into 3 groups based on their diet (NPD and HFD) and on GTJ treatment, continuing until T2, which was 5 weeks after T1.

Specifically, the normal control group was fed with a normal diet (NPD, n = 6) until T2, while the second one (HFD group, n = 6), serving as the MetS control, was fed with the HFD throughout the trial (from T0 to T2). The control NPD and HFD groups were subjected to the same stress conditions as the treated group since they received, during the last month of the experiment, from T1 to T2, a volume of vehicle (plain water) equal to the GTJ solution administered to the treated group. Indeed, one group (HFD-GTJ, n = 8) was treated with 2 mL of GTJ daily in the final month of the trial (T1–T2).

#### 2.3.2. Preparation of the Orally Administered Tomato Solutions

The amount of GTJ administered daily for 5 weeks to the treatment group of animals (HFD-GTJ) was 2 mL/kg body weight, distributed in two administrations of no more than 1 mL each. Taking into account the concentration of 9-oxo-10(E),12(E)-ODA present in 1 mL of extract is equal to 0.22 µg, corresponding to 10 mg of lyophilizate, which is equivalent to 125 mL of GTJ, the amount of 9-oxo-10(E),12(E)-ODA taken daily by the animal corresponds to 0.014 µg in 2 mL. To relate the amount of juice fed to the animal to an individual weighing 70 kg, the amount of juice to be taken daily should be 140 mL, a dose that can easily be taken as a snack.

### 2.4. Experimental Design

In T1, after 8 weeks of HFD, the induction of the metabolic syndrome was confirmed by demonstrating an increase of at least 70% in at least three biochemical parameters, including body weight, triglycerides, cholesterol, and LDL, as reported in previous studies [11].

At the T2 time point, the impact of treatment on the experimental groups was assessed for MetS-induced alterations in biometric, biochemical, and oxidative homeostasis parameters. At the conclusion of the experimental procedures, all animals were sacrificed using 2% isoflurane anesthesia, followed by cervical dislocation, in accordance with authorized protocols. Plasma samples were collected for subsequent analyses to evaluate lipid and glucose homeostasis, oxidative stress parameters, and plasma antioxidant status. Hepatic samples were also collected for malondialdehyde (MDA), as well as reactive oxygen and nitrogen species (RONS) and determination for histological evaluations.

#### 2.4.1. Biometric Parameters and Leptin Levels

Biometric parameters such as body weight were monitored throughout the experiment. Importantly, body weight (g) per rat was evaluated at T2 for all animals, after 5 weeks of nutritional treatments, to assess any potential changes among groups. We also evaluated food intake (g per rat), as previously detailed [11]. Furthermore, plasma leptin levels were analyzed using plasma collected from blood samples obtained from the animals post sacrifice at time point T2. The blood was centrifuged at 2000 rpm for 15 min at room temperature, with the brake function disabled to prevent hemolysis. Leptin levels were measured via enzyme immunoassay, utilizing the ELISA kit provided by Millipore, Saint Louis, MO, USA. Plasma samples were diluted 1:3, and leptin concentrations were quantified using a calibration curve ranging from 10.97 to 8000 pg/mL, with spectrophotometric readings at a wavelength of 450 nm. The kit’s detection sensitivity was 30 pg/mL.

#### 2.4.2. Histological Analysis and PPAR-α Expression by Real-Time PCR of Hepatic Tissue

Hepatic samples were fixed in formalin and embedded in paraffin. Sections (5 μm) were obtained from paraffin blocks of samples with a cutting microtome and stained with hematoxylin and eosin for histological evaluation. Following staining, the slides were examined with an optical microscope (Microscope Axioscope 5/7 KMAT, Carl Zeiss, Oberkochen, Germany) connected to a digital camera (Microscopy Camera Axiocam 208 color, Carl Zeiss). Steatosis was evaluated using semiquantitative analysis performed by two independent observers in a high-power field (HPF) (magnification 400×) and repeated for 10 HPFs in n = 4 samples per experimental group.

Additionally, we evaluated hepatic mRNA levels of PPAR-α expression by Real-Time PCR. In detail, 30 µg of rat liver was homogenized in 300 µL of QIAzol Lysis Reagent (Cat. No. 79306, Qiagen, Hilden, Germany), and RNA was isolated using the Invitrogen PureLink RNA Mini kit (Catalog number:12183018°, Invitrogen by Thermo Fisher Scientific) following the manufacturer’s instructions. MultiskanGO Microplate Spectrophotometer (Thermo Scientific, Waltham, MA, USA) was used to evaluate the RNA concentration. A total of 2 µg of RNA was immediately reverse transcribed using the High Capacity cDNA Reverse Transcription Kit (4368814, Thermo Fisher Scientific). The reaction mixtures (20 µL) were incubated for 10 min at 25 °C, 120 min at 37 °C, and then for 5 min at 85 °C. The obtained cDNA was subjected to a SYBR Green-based Real-time PCR performed in 48-well plates using the Step-One Real-Time PCR System (Applied Biosystems, Waltham, MA, USA). The real-time reaction mixture containing 2X SYBR Green Master Mix (A46109, Applied Biosystems), 2 µL of cDNA, and 0.6 μM of forward and reverse primers (PPAR-alpha-FW: 5′-ACGATGCTGTCCTCCTTGATG-3′, PPAR-alpha-Rev: 5′-GCGTCTGACTCGGTCTTCTTG-3′; β-actin-FW: 5′-AAGGCCAACCGTGAAAAGAT-3′, β-actin-Rev: 5′-TGGTACGACCAGAGGCATAC-3′) was amplified with the following protocol: 95 °C for 20 s, followed by 40 cycles of 95 °C for 3 s and 60 °C for 30 s. The levels of the target gene were normalized to β-actin levels, and its relative changes in expression were calculated as 2^−ΔCt^ (ΔCt = Ct gene of interest − Ct internal control) [26].

#### 2.4.3. Metabolic Assays: Glucose Tolerance and Lipid Homeostasis

For evaluation of glucose homeostasis, before sacrifice, we performed the glucose tolerance test (GTT), a diagnostic tool for diabetes and an indicator of metabolic efficiency and insulin resistance, according to established procedures [11,27]. In the plasma samples obtained at T2, concentrations of triglycerides (TG), total cholesterol (TC), Low-Density Lipoprotein cholesterol (LDL), and High-Density Lipoprotein cholesterol (HDL) were measured using commercial kits with the Free Carpe Diem device (FREE^®^ Carpe Diem; Diacron International, Italy), as detailed previously [11]. The data are consistently expressed in mg/dL.

#### 2.4.4. Systemic and Hepatic Redox Homeostasis Parameters

Plasma redox balance was evaluated by Diacron kits using the Free Carpe Diem device (FREE^®^ Carpe Diem; Diacron International, Italy), following detailed procedures previously published [11]. To assess the pro-oxidant status, the dROMs (Reactive Oxygen Metabolites, primarily hydroperoxides) and the LP-CHOLOX (Lipoperoxides and Oxidized Cholesterol) tests were performed. The dROMs test measured the levels of hydroperoxyl free radicals, while the LP-CHOLOX test assessed the levels of circulating lipid peroxides and, in particular, oxidized cholesterol. Data from the dROMs test are expressed in arbitrary units, specifically Carratelli units (UCARR). The normal range of the test results was 250–300 U.CARR (Carratelli Units), where 1 U.CARR corresponds to 0.08 mg/dL of H_2_O_2_ [28]. In the LP-CHOLOX Test, lipoperoxides and oxidized cholesterol levels are detected based on peroxides’ ability to facilitate the oxidation of Fe^2+^ to Fe^3+^ binding to an indicator mixture to form a colored complex detected by a spectrophotometer at 505 nm [29,30]. The results are expressed in mEq/L.

Regarding plasma antioxidant status, the BAP-TesT (Biological Antioxidant Potential) assesses exogenous substances (ascorbate, tocopherols, carotenoids, and bioflavonoids) and endogenous substances (bilirubin, uric acid, and proteins) with an antioxidant potential capable of counteracting radical species in the plasma. The analysis was conducted using the Diacron kit with spectrophotometric readings at a wavelength of 505 nm, and results are expressed in mmol/L, as described in previous studies [31]. Additionally, the SHp test was used for the evaluation of thiol groups, assessing the reducing properties of GTJ that can counteract thiol group oxidation and shift the balance towards reduced forms.

##### MDA Evaluation in Hepatic Tissue

Assessment of MDA levels in liver homogenates was conducted following the method by Ohkawa et al. [32]. In brief, the reaction mixture included 0.2 mL of whole homogenate, 0.2 mL of 8.1% sodium dodecyl sulfate (SDS), and 1.5 mL of acetic acid solution adjusted at pH 3.5 with NaOH and 1.5 mL of 1% thiobarbituric acid (TBA) aqueous solution. The mixture was then brought to a total volume of 4.0 mL with distilled water and heated at 95 °C for 60 min. After cooling with tap water, 1.0 mL of distilled water and 5.0 mL of a n-butanol/pyridine solution (15/1, *v*/*v*) were added, and the mixture was shaken vigorously. Following centrifugation at 4000 rpm for 10 min, the absorbance of the organic layer was measured at 532 nm by using a plate reader (GloMax Plate Reader, Promega, Milan, Italy). MDA levels are expressed as nmol MDA/g tissue, using 1,1,3,3, tetramethoxypropane as an external standard.

##### RONS Evaluation in Hepatic Tissue

RONS levels were assessed in liver homogenates using 2′,7′dichlorodihydrofluorescein diacetate (H2DCF-DA), as previously reported [33]. In brief, whole homogenates were centrifuged at 3500 rpm for 10 min at 4 °C, and 100 µL of the supernatant was mixed with 5 µL of H2DCF-DA at a final concentration of 10 µM. The reaction mixture was incubated for 30 min at 37 °C, protected from light, and the fluorescence intensity was detected at 490 nm (excitation) and 540 nm (emission) using a plate reader (GloMax Plate Reader, Promega, Milan, Italy).

### 2.5. Statistical Analyses

Statistical analysis was performed using GraphPad Prism 9.02 (San Diego, CA, USA). Differences in the nutritional composition of GTJ and GT were analyzed by an unpaired Student’s *t*-test. The results are expressed as mean ± standard deviation of three repetitions.

Body weight and plasma glucose levels in the GTT were analyzed via a two-way repeated measures (RM) ANOVA, followed by Bonferroni post hoc test for significant differences for within- and between-subject comparisons, considering the effect of “time”, “diet”, and their interaction in the experimental groups. Values of body weight, TG, TC, LDL, HDL, AUC, MDA, RONS, leptin, and redox homeostasis parameters levels and histological evaluations in liver were compared by a one-way ANOVA test followed by Bonferroni post hoc evaluations for differences between means. The data were represented by scattered graphs showing the mean ± standard error of the mean (S.E.M.), including at least 4 animals per group. Differences were considered significant when *p* < 0.05. The statistical power (g-power) was considered only if >0.75 and the effect size if >0.40.

## 3. Results

### 3.1. Morphological Characteristics of Tomato Plants

Sixty days after transplanting, the plants reached a height of 80 cm, covering an average surface area of approximately 1 m^2^. Flowering began 18 days after transplanting, while the first fruit set on the first truss was detected 26 days after transplanting. Five fruits were observed on the first truss, on average, with a fruit set percentage close to 80%. The yield of the fruits harvested at full ripening was 8.83 ± 0.12 kg/plant. The average yield harvested to produce golden juice (classes 2, 3 and 4) was 9.8 ± 0.12 kg/plant, with an increase of about 11%.

### 3.2. Chemical and Nutritional Properties of Golden Tomatoes After Juice Processing

GTJ has similar nutritional characteristics to GT, except for a reduced protein level that makes the juice lower in calories than the fruit. On the other hand, as far as micronutrients are concerned, juice processing decreases the content of mineral salts, organic acids, and vitamin C (74% less), while vitamin A is slightly increased. The chemical and nutritional parameters of GTJ compared with those of the GT are given in Table 2A,B below.

Citric acid, triprotic, accounts for 30% of the dry matter, and malic acid, biprotic, for 4% of the dry matter. The processing of Golden Tomatoes into juice results in a 30% reduction in citric acid, 35% in malic acid, and a total loss of tartaric acid, which is present in traces. Oxalic acid has a marginal effect on dry matter (0.4%) and shows a 19% reduction during processing compared with tomatoes. Glutamic acid, on the other hand, is formed during the processing of the juice and, unlike the Golden Tomato, is present and contributes 2% to the dry residue.

Regarding the micronutrients of GTJ shown in Table 2C, taking into account the Recommended Dietary Allowances (RDAs) given by the World Health Organization (WHO), calculated for healthy adults, and comparing the micronutrient values obtained in 100 g of tomato juice with the RDAs, its low sodium content emerges (0.72%/100 g RDA). It is also a good source of iron for men (8.4%/100 g of RDA) and women (4.6%/100 g of RDA), as well as vitamin C (7.3%/100 g of RDA), and it is a good source of magnesium (3.6%/100 g of RDA), potassium (3.8%/100 g of RDA), and copper (2%/100 g of RDA), while it is low in calcium (1.1%/100 g of RDA), zinc, for both women (1.1%/100 g of RDA) and men (1.3%/100 g of RDA), and manganese (0.7%/100 g of RDA). Furthermore, its low zinc content of 0.2 mg/100 g makes it well tolerated by those with metal intolerance.

### 3.3. Fatty Acid Content “9-Oxo-10(E),12(E)-ODA” in GTJ Compared with Red and Golden Tomatoes

Qualitative and quantitative analyses were carried out on both fully ripe (red) and veraison stage (golden) tomatoes, as well as on the derived Golden Tomato Juice. Attention was focused on the isomer 9-oxo-10(E),12(E)ODA.

**Figure 2:**
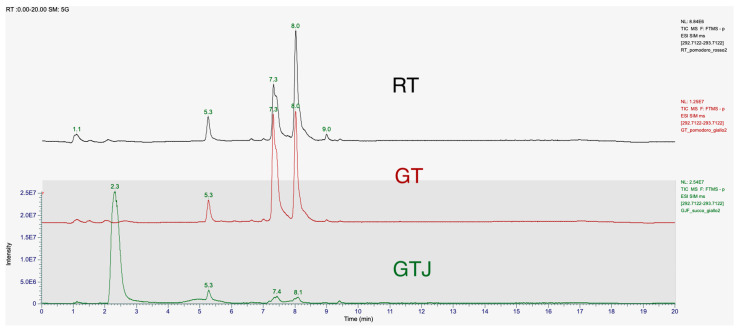
T-SIM mode chromatogram of 9-oxo-10(E),12(E)-ODA at a concentration of 1 μg/mL in samples of freeze-dried red tomato (RT) extract, freeze-dried Golden Tomato (GT) extract, and freeze-dried tomato juice (GTJ) extract. The first elution peak is 9-oxo-10(E),12(Z)-ODA, T.R. 7.4 min, and the next peak is 9-oxo-10(E),12(E)-ODA, T.R. 8.1 min. The results are expressed as mean ± SD of two replicates.

The chromatographic spectra of the tomato extracts in Figure 2, red golden, and Golden Tomato Juice, showed two peaks at retention times of 7.4 min and 8.1 min. The peak of our 9-oxo-10(E),12(E) octadecadienoic acid standard corresponds to the peak at the retention time of 8.1 min. The first peak may correspond to the isomer 9-oxo-10(E),12(Z) octadecadienoic acid (9-oxo-10(E),12(Z)-ODA), as reported in the literature [21]. These two stereoisomers differ in having their hydroxy residues in the *S* or *R* configurations. The retention times (R.T.) and relative concentrations of the isomers detected in the different matrices, tomato and processed, at a concentration of 1 μg/mL, are shown in Table 3 with the statistical significance and mean values of concentration.

In detail, both isomers are present in higher concentrations in GT than in RT, the 9-oxo-10(E),12(E)-ODA isomer being present in 27% higher concentrations than in red tomato, while the 9-oxo-10(E),12(Z)-ODA isomer is present in 57% higher concentrations. In tomato juice, both isomers are present in significantly lower concentrations than in Golden Tomato, 79% for the 9-oxo-10(E),12(E)-ODA isomer and 84% for the 9-oxo-10(E),12(Z)-ODA isomer, respectively. Indeed, one-way ANOVA revealed that GT contains more 9-oxo-10(E),12(E)-ODA than RT at both retention times considered (respectively, F_(2,3)_ = 347.5, *p* = 0.0003 and F_(2,3)_ = 385.8, *p* = 0.0002).

### 3.4. Parameters Assessed at T1 Time to Verify the Induction of Metabolic Syndrome

At T1, following 8 weeks of high-fat diet (HFD), an increase of at least 70% in at least three biochemical or biometric parameters was found in the HFD group vs. normal-fed rats, confirming the onset of the metabolic syndrome, as reported in previous studies [10,11]. Indeed, body weight was analyzed together with biochemical parameters from representative plasma samples such as TG and TOT Chol. In particular, significant differences in HFD vs. NPD were found at T1 on the following parameters, i.e., body weight (*p* = 0.0230, t = 3.590, df = 4), triglycerides (*p* = 0.0003, t = 11.34, df = 4), and total cholesterol (*p* = 0.0144, t = 4.139, df = 4).

### 3.5. Effects of GTJ Treatment on Body Weight, Food Intake, and Leptin Levels in MetS

**Figure 3:**
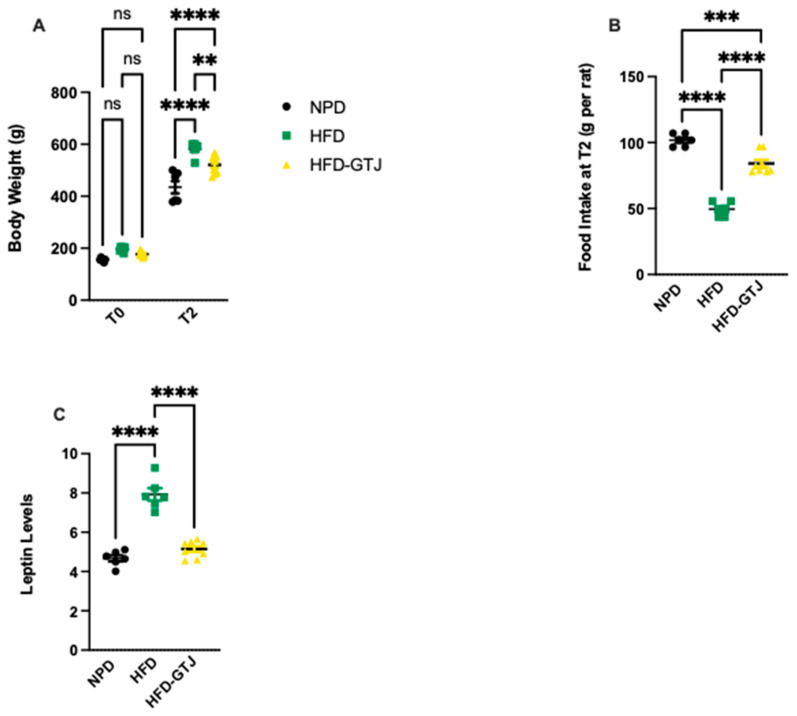
(**A**) Variation in body weight from T0 to T2 (g) in HFD-GTJ, HFD, and NPD experimental groups. Statistical significance by two-way ANOVA followed by post hoc Bonferroni is indicated as (**) *p* < 0.01, (****) *p* < 0.0001, and (ns) not significant. (**B**) Variation in food intake at time T2 in NPD, HFD, and HFD-GTJ groups. Statistical significance by one-way ANOVA followed by post hoc Bonferroni is indicated as (***) *p* < 0.001 and (****) *p* < 0.0001. (**C**) Leptin levels in NPD, HFD, and HFD-GTJ groups. Statistical significance by one-way ANOVA followed by post hoc Bonferroni is indicated as (****) *p* < 0.0001.

In T0, the results from body weight show that all animals are subdivided into homogenous groups. At the end of the experiment (T2), the HFD group shows an average 25% increase in body weight compared with the NPD group, with associated increased leptin levels leading to reduced food intake compared with the NPD group, as can be seen in Figure 3. Treatment with GTJ resulted in a lower average body weight by 10% in the treated group (HFD-GTJ) compared with the HFD group. At the same time, the lower values of body weight are also associated with significantly lower leptin levels in the HFD-GTJ group compared with the HFD group but not compared with the NPD group. The lower leptin levels are associated with an increase in food intake in the HFD-GTJ, unlike that observed in the HFD group. In detail, significant differences in body weight were found by two-way ANOVA between NPD, HFD, and HFD-GTJ considering time (F_(1,34)_ = 1188, *p* < 0.0001), nutritional treatment (F_(2,34)_ = 28.65, *p* < 0.0001), and their interaction (F_(2,34)_ = 9.19, *p* = 0.0006), as in Figure 3A. Furthermore, one-way ANOVA was conducted on food intake at T2 in the experimental groups and outlined significant differences between groups (F_(2,17)_ = 100.7, *p* < 0.0001), as in Figure 3B. A one-way ANOVA followed by a Bonferroni post hoc test showed that leptin levels were elevated in the HFD group compared with the NPD group. However, following GTJ supplementation, HFD rats showed significantly lower leptin levels versus HFD alone and were not different from NPD control values in the HFD-GTJ group (F_(2,17)_ = 64.06; *p* < 0.0001, Figure 3C).

### 3.6. Effects of GT Juice in the Liver on Steatosis and PPAR-α Levels in MetS

**Figure 4:**
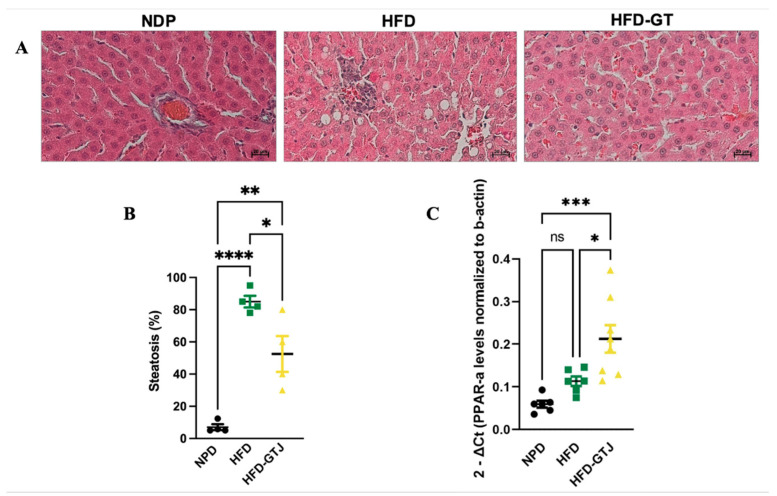
(**A**) Histological features of liver tissue of experimental groups: NPD; HFD; HFD-GTJ. Representative images of hematoxylin and eosin staining of liver tissue: magnification 200×, scale bar 50 µm. (**B**) Histological evaluation of hepatic steatosis. Differences in hepatic steatosis (%) between NPD, HFD, and HFD-GTJ groups. Statistical significance of Bonferroni post hoc tests is indicated for (*) *p* < 0.05, (**) *p* < 0.01, and (****) *p* < 0.0001, as represented in the graphs. (**C**) Peroxisome proliferator-activated receptor alpha (PPAR-α) expression in HFD-GTJ, NPD, and HFD groups. The PPAR-α expression was expressed as 2^−ΔCt^, where ΔCt is (Ct gene of interest − Ct housekeeping gene). β-Actin was used as a housekeeping gene. Statistical significance by one-way ANOVA followed by post hoc Bonferroni is indicated as (*) *p* < 0.05, (***) *p* < 0.001, and (ns) not significant.

The histological analysis of liver samples from the control NPD group revealed minimal steatosis, with an average percentage of 7.03 ± 3.59, in contrast to the HFD group, where steatosis was significantly elevated, averaging 85 ± 7.25. In the HFD liver tissue, macrovesicular steatosis characterized by large lipid droplets was primarily observed. Conversely, liver samples from the HFD-GTJ group exhibited microvesicular steatosis, with an average percentage of 52.5 ± 22.17. Statistical evaluation by one-way ANOVA revealed significantly lower levels in the percentage of steatosis in the HFD group after GTJ supplementation compared with the HFD group, although it remained significantly higher than in the NPD group (F_(2,9)_ = 33.02, *p* < 0.0001, Figure 4A,B). Furthermore, a one-way ANOVA on liver peroxisome proliferator-activated receptor alpha (PPAR-α) expression revealed notable differences between groups (F_(2,17)_ = 11.27, *p* = 0.008, Figure 4C). In detail, the HFD-GTJ group showed a marked increase in expression of PPAR-α of 47% compared with both the HFD group and the NPD group. No significant differences emerged from the post hoc test between the HFD and NPD groups.

### 3.7. Metabolic Effects of GT Juice in MetS: Glucose Tolerance and Lipid Profile

**Figure 5:**
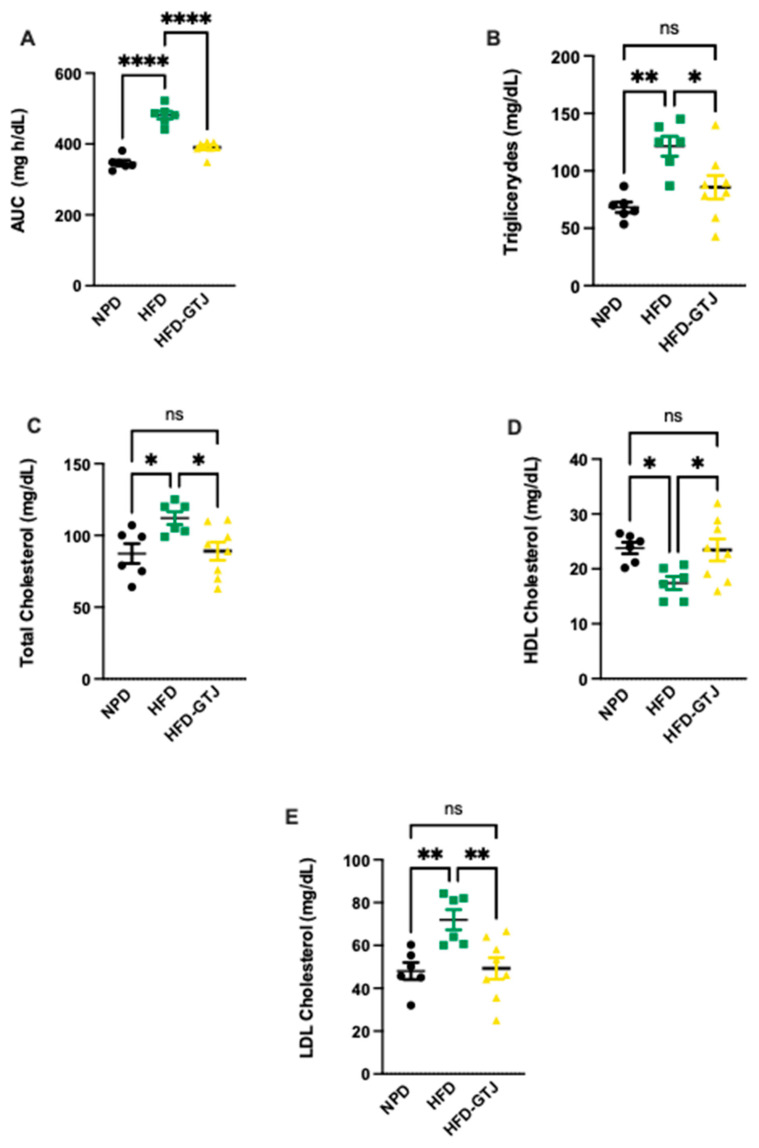
Metabolic parameters of glucose and lipid homeostasis: (**A**) Area under the curve (AUC) of plasma glucose levels (mg/dL) per unit of time (h) difference between NPD, HFD, and HFD-GTJ groups. Statistical significance by one-way ANOVA followed by post hoc Bonferroni is indicated as (****) *p* < 0.0001. (**B**) Total cholesterol, (**C**) LDL cholesterol, (**D**) HDL cholesterol, and (**E**) triglycerides (mg/dL) in HFD-GTJ, HFD, and NPD experimental groups. Statistical significance by one-way ANOVA followed by post hoc Bonferroni is indicated as (*) *p* < 0.05, (**) *p* < 0.01, and (ns) not significant.

Regarding the metabolic effects of GTJ, a glucose tolerance test (GTT) was conducted in T2, revealing significant differences between the experimental groups. In detail, we assessed the area under the curve (AUC) using a one-way ANOVA followed by a Bonferroni post hoc test, which showed significantly lower levels in the HFD-GTJ group compared with the HFD group, returning to control levels similar to the NPD group (F_(2,17)_ = 62.43, *p* < 0.0001, Figure 5A). This indicates an improvement in glucose tolerance following GTJ supplementation. As regards lipid profile, in T2, plasma concentrations of triglycerides (TG), total cholesterol (TC), Low-Density Lipoprotein (LDL), and High-Density Lipoprotein (HDL) cholesterol were compared among the HFD-GTJ, HFD, and NPD groups. A one-way ANOVA on total cholesterol levels in the plasma revealed significant differences between groups, with substantially higher levels observed in the HFD group compared with the NPD group and the HFD-GTJ group, which was non-significant vs. NPD (F_(2,17)_ = 4.68, *p* = 0.0239, Figure 5B). Regarding LDL cholesterol, a significant decrease was shown in the HFD-GTJ group compared with the HFD group (F_(2,17)_ = 7.52, *p* = 0.0046, Figure 5C). Furthermore, the analysis of HDL levels by one-way ANOVA showed significantly higher values in the HFD-GTJ group following GTJ treatment compared with the HFD group, with levels similar to NPD values (F_(2,17)_ = 4.53, *p* = 0.0265, Figure 5D). Notably, triglyceride levels were reduced by GTJ supplementation in the HFD-GTJ group, reaching significantly lower levels than in the HFD group, with no difference compared with the NPD group (F_(2,17)_ = 8.63, *p* = 0.0026, Figure 5E).

### 3.8. Effects of GT Juice on Systemic and Hepatic Redox Homeostasis in MetS

**Figure 6:**
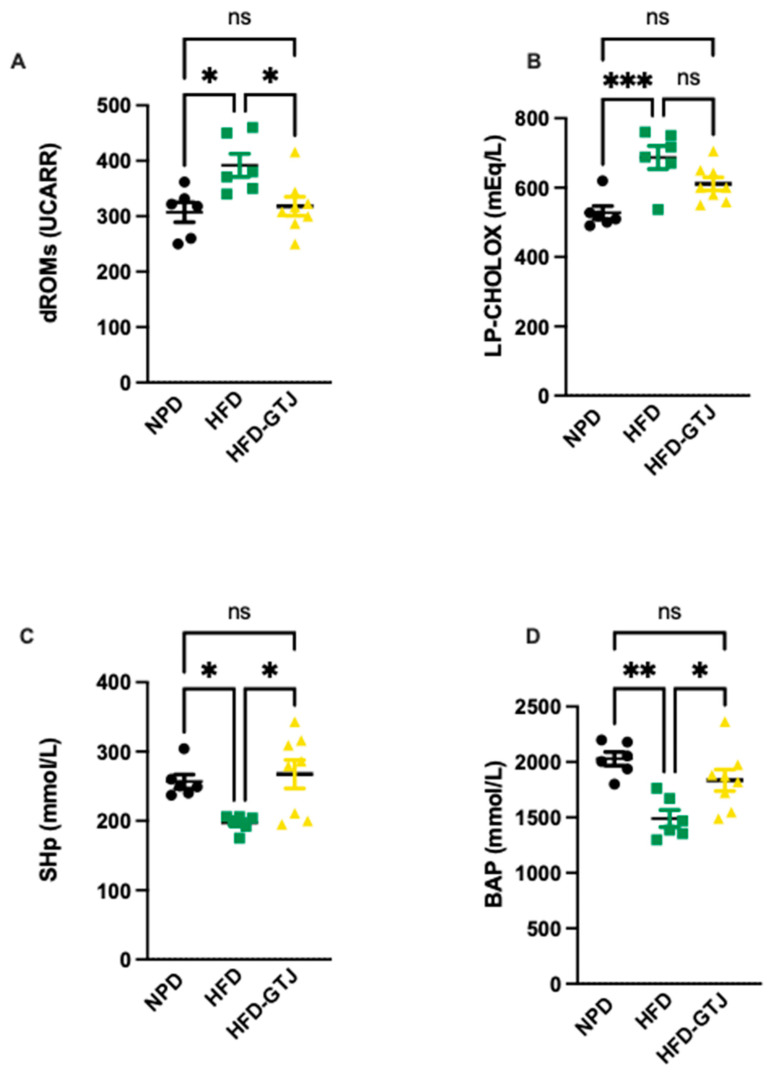
Plasma redox homeostasis biomarkers between NPD, HFD, and HFD-GTJ groups at the end of experimental procedures. Pro-oxidant status evaluated by (**A**) dROMs test for differences in ROM (primarily hydroperoxide) levels (UCARR) and (**B**) LP-CHOLOX test for differences in LP-CHOLOX (lipoperoxides and oxidized cholesterol) levels (mEq/L). Antioxidant status evaluated by (**C**) SHp test for thiol group levels (mmol/L) and (**D**) Biological Antioxidant Potential (BAP test) levels (mmol/L). Statistical significance of Bonferroni post hoc tests is indicated by (*) *p* < 0.05, (**) *p* < 0.01, (***) *p* < 0.001, and (ns) not significant, as represented in the graphs.

**Figure 7:**
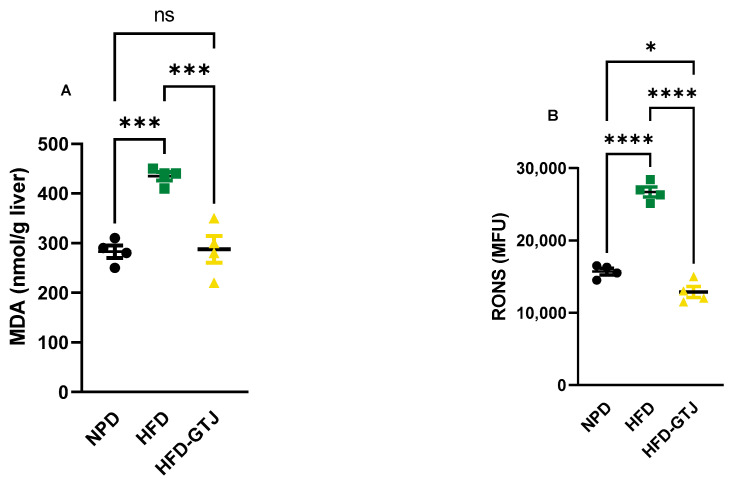
MDA (**A**) and RONS (**B**) levels in NPD, HFD, and HFD-GTJ groups. Statistical significance by one-way ANOVA followed by post hoc Bonferroni is indicated as (*) *p* < 0.05, (***) *p* < 0.01, (****) *p* < 0.001 and (ns) not significant.

The antioxidant and pro-oxidant status was evaluated in T2, after the nutritional intervention with GTJ, using plasma samples from all experimental groups to examine the redox balance in MetS. Significant differences were found in pro-oxidant status, particularly in dROMs and LP-CHOLOX levels, indicating that GTJ supplementation helped counteract the changes induced by HFD and showed values similar to the NPD control group. Specifically, dROM levels varied among the experimental groups, with post hoc analysis revealing significantly lower levels in the HFD-GTJ group compared with the HFD group (F_(2,17)_ = 5.731, *p* = 0.0125; Figure 6A). Additionally, a one-way ANOVA for LP-CHOLOX levels showed significant differences among the three groups, but post hoc analysis indicated that the NPD group had lower levels than the HFD group, which were not significantly different from those in the HFD-GTJ group (F_(2,17)_ = 10.26, *p* = 0.0012; Figure 6B). Furthermore, statistical analysis demonstrated a significant impact of GTJ on the antioxidant capacity in MetS animals. Specifically, a one-way ANOVA followed by post hoc testing showed that SHp values were significantly higher in the HFD-GTJ group compared with the HFD group and not significantly different from the control group (F_(2,17)_ = 5.817, *p* = 0.0119; Figure 6C). Similarly, BAP values were elevated in the HFD-GTJ group compared with the HFD group, with no significant difference from the NPD group (F_(2,17)_ = 9.474, *p* = 0.0017; Figure 6D). At the hepatic level, evaluation of the liver redox state was then carried out by assessing both MDA and RONS levels. As shown in Figure 7, the HFD group showed a marked and significant increase in the levels of both MDA (A) and RONS (B) when compared with the NPD ones (respectively, F_(2,9)_ = 23.60, *p* = 0.0003 and F_(2,9)_ = 125.0, *p* < 0.0001). Remarkably, administration of GT juice showed lower MDA levels similar to control ones and reduced the RONS ones below the values of the NPD group.

## 4. Discussion

Tomato fruit *(Lycopersicon esculentum* Mill.), a component of the Mediterranean diet, has gained increasing attention as it is widely cultivated and consumed globally, serving as an invaluable source of bioactive compounds [34]. The huge plethora of nutritional substances encountered in this food is remarkable, i.e., antioxidants, macronutrients, micronutrients, and organic and phenolic acids. The composition and quantity of these molecules vary depending on the cultivating conditions, being able to influence health-promoting activities, though this aspect still requires comprehensive biochemical and nutritional characterization. Data in the literature have highlighted its beneficial properties due to the wealth of bioactive components in its matrix, which is able to counteract dyslipidemia [35,36], type II diabetes, neurodegeneration [37], cardiovascular disease, and certain cancers [38].

Also, tomato-based products may have protective roles, such as modulating lipid profiles and positively influencing the development of cardiovascular diseases [39,40,41,42]. Among them, previous findings on the GT in the model of MetS encouraged us to investigate a further derivative product, i.e., the juice, that can be obtained from the GT. In the present study, we observed that GTJ—compared with the tomato from which it is derived—has good nutritional characteristics, such as low sodium and good iron content, which, together with its low energy value, make it a good candidate as part of a balanced diet. In addition, the juice has a lower total acidity and reduced levels of citric acid, which is a triprotic acid responsible for rounder flavor [43,44]. The transformation process results in the loss of proteins and the formation of free amino acids, such as glutamic acid, which give the juice an umami flavor, making it more palatable to the consumer [45]. The presence of glutamic acid improves the salty taste without increasing the salt content; this is in line with the World Health Organization’s requirement to reduce hypertension, an important risk factor for mortality [43]. The presence of organic acids in the juice, in addition to influencing the flavor, improves its nutritional properties. In fact, malic acid in the form of exogenous malate is easily absorbed and is able to integrate the activity of malate dehydrogenase, maintaining high levels of the intermediates of the tricarboxylic acid (TCA) cycle by increasing the speed of the TCA and the malate–aspartate shuttle, influencing the body’s energy requirements [46]. Another relevant feature of GTJ is the presence of the fatty acid 9-oxo-10(E),12-(E)-octadecadienoic acid, which, as already found in red tomato juice due to its role as a potent activator of PPAR alpha, is able to reduce triglyceride accumulation in primary rat hepatocytes [47]. The reduced presence of the 9-oxo-10(E),12(E)-ODA isomer in the GTJ compared with fresh tomato fruits could be related to its conversion to the 13-oxo-10(E),12(E)-ODA isomer as a result of the transformation process into juice [21].

After the insight gained on GTJ in the first part of this study, we explored the effects of the oral administration of GTJ to HFD rats for 5 weeks.

Regarding the effects of GTJ supplementation on body weight and leptin-mediated appetite control in the MetS model, our results show that GTJ is able to positively influence these parameters. Supplementation with GTJ is able to slow down the rate of weight gain induced by the HFD, which is confirmed to be higher than in the normo-caloric diet (NPD group). Similarly, it can be observed that the HFD-GTJ group has lower levels of blood leptin levels compared with the HFD, which is consistent with the lower body weight observed. Interestingly, as the inhibitory effect of leptin as a regulator of satiety is reduced, more hyperphagia is observed in juice-treated rats than in HFD rats. Leptin, a hormone produced by adipose tissue, plays a crucial role in regulating appetite and energy balance [48]. The lower leptin levels observed in the GTJ-treated group compared with the HFD, combined with the higher food intake vs. HFD, suggest that the juice may induce less satiety associated with a lower adiposity-associated hyperleptinemia. This could partially suggest that the increase in food intake in the HFD-GTJ group, without a corresponding increase in body weight, could be a consequence of the modulation of leptin levels, which may have helped recalibrate energy expenditure mechanisms. The observed food behavior is in agreement with data from Zhao et al., showing that in genetically modified mice, reduced levels of leptin are protective against HFD-induced obesity [49].

To deepen our knowledge of GTJ at the tissue level in MetS, a crucial parameter to evaluate is the histological assessment of the liver. Indeed, we observed that in the liver of HFD rats, microvesicle droplets were increased versus NPD controls, which is linked to hepatic steatosis and NAFLD. Interestingly, GTJ treatment was able to reduce lipid droplets in the liver parenchyma of HFD rats.

Once assessed the amelioration of steatosis in the HFD-GTJ group, we investigated the expression of PPAR-α mRNA levels in the hepatic parenchyma. Our outcomes revealed that after supplementation with GTJ, we observed higher levels of the expression of mRNA coding for PPAR-alpha in the liver tissue compared with HFD and NPD. Considering the effects reported in the literature of the isomers 9-oxo-ODA and 13-oxo-ODA on PPAR-alpha expression in the liver, and given the presence of the isomer 9-oxo-10(E), 12(E)-ODA in the juice, we can speculate on its possible contribution to the mechanism. This may be a clue for future investigations. However, HFD alone does not influence hepatic PPAR-α levels with respect to NPD, hence suggesting that it is not the diet protocol itself that modulates the PPAR-α pathway, but it could be a specific response associated with the GTJ treatment [50]. PPAR-α is known to play a crucial role in regulating the metabolic adaptation to increased fatty acids. Its activation helps maintain hepatic lipid homeostasis by regulating catabolic and synthetic pathways, thereby limiting the cytotoxic effects of free fatty acids (FFAs) [8]. The presence of the specific PPAR-α agonist, i.e., 9-oxo-10(E),12(E)-ODA, that we found in the GTJ could be implicated in the alteration of mRNA hepatic levels of PPAR-α in the HFD-GTJ group putatively by inducing an upregulation, as already found in hepatocytes [47]. In addition to that, we should consider that in the HFD, the elevated plasma FFAs—known as endogenous PPARα ligands during fasting and to mediate the fasting-induced metabolic effects—are not able to modify hepatic PPARα expression [50,51].

The regulatory action performed by GTJ in the HFD model has also been evidenced in metabolism in terms of glucose tolerance and lipid profile. In our study, we observed an improved glucose tolerance that was altered by the hyperlipidemic diet following GTJ administration in HFD rats. This may be due to the modulation of insulin sensitivity played by PPAR-α [8], since PPAR-α activation ameliorates insulin signaling, for instance, via improvement of peripheral utilization of glucose and glycogen storages [52]. Indeed, in the presence of PPAR-α ligands, glucose binds to PPAR-α and attracts coactivators [53]. However, the pathways by which activation of PPAR-α transcription by fatty acid is able to influence glucose metabolism remain to be investigated; among them, it could be the increase in GLP-1 levels via PPAR-α-dependent and -independent pathways [54]. The assessment of GLP-1 levels could therefore constitute a future direction of our research.

As for lipid metabolism, we outlined an improvement in lipid homeostasis in HFD-GTJ rats, in terms of lower levels of triglycerides, LDL, and total cholesterol, with a concomitant increase in HDL, which could be due to the activation of PPAR-α by GTJ, enhancing fatty acid oxidation and improving lipid profiles. Indeed, PPAR-α increases the expression of cytochrome P4504A (CYP4A), which catalyzes the ω-hydroxylation of fatty acids and contributes to the reduction in TG synthesis [19]. In addition, activation of PPAR-α by 9-oxo-10(E),12(E)-ODA present in GTJ probably stimulates the production of key enzymes such as carnitine palmitoyltransferase 1 (CPT1) and acyl-CoA dehydrogenase, which are essential for fatty acid catabolism in the liver, thus reducing lipid accumulation [19,55]. In accordance with this, PPAR-α agonists are already used to treat dyslipidemia, a condition marked by decreased triglyceride levels and increased HDL-c levels in the blood plasma [56]. This effect can be achieved by boosting the production of the main components of HDL-c, known as apolipoproteins A-I and A-II (APO A-I and APO A-II) [8,57], which play a crucial role in the reverse cholesterol transport (RCT) pathway from peripheral cells. The proposed mechanisms through which PPAR-α reduces plasma triglycerides include enhanced hepatic expression of lipoprotein lipase (LPL) and the inhibition of APO CIII in the liver [8].

It should be mentioned that the observed effects on lipid and glucose metabolism could also be a consequence of the action of leptin. It appears that leptin acts indirectly by reducing the lipogenic effects of insulin [58].

Regarding redox homeostasis, GTJ supplementation influences systemic redox balance, since we observed lower levels of reactive oxygen species, such as hydroperoxides (dROMs), compared with physiological levels in HFD rats, while enhancing antioxidant defenses, as evidenced by a higher endogenous antioxidant barrier, i.e., BAP test and increased thiol groups. This could be a consequence of the activation of PPAR alpha, since it is known to play a key role in reducing oxidative stress by activating antioxidant enzymes, such as heme oxygenase [59], superoxide dismutase [60], and thyroxine reductase [60,61].

Translating the GTJ effect on systemic redox balance to liver oxidative stress, our results clearly show that GTJ administration significantly counteracts hepatic oxidative stress, showing MDA levels similar to control levels and RONS ones even below NPD levels. These data could be ascribed to the PPAR-α pathway, consistently with GTJ effects on lipid catabolism, since PPAR-α increases the expression of uncoupling proteins that help maintain intracellular redox homeostasis by decreasing mitochondrial RONS production [62,63]. In this scenario, the regulation of PPAR-α activation is closely linked to the modulation of the cellular oxidative state. By activating PPAR-α, the bioactive compound present in the GTJ could modulate the cellular response to oxidative stress and influence metabolic pathways. Notwithstanding this, the effects of GTJ on antioxidant properties can also be attributed to the action of the complex of phytonutrients present with their antioxidant properties, thus deserving further research.

## 5. Conclusions

In conclusion, GTJ emerges as a promising functional food that can offer beneficial nutritional properties, such as low sodium and rich iron content. Importantly, GTJ supplementation in MetS led to lower levels of body weight, improvements in glucose tolerance, and enhanced lipid profiles. Additionally, GTJ’s ability to influence systemic leptin levels highlights its potential in weight management by positively reinforcing the metabolic effects observed. All these health-promoting effects could be linked to the activation of PPAR-α, since we observed higher hepatic mRNA levels in HFD rats that were treated with GTJ. In this context, we found that among the bioactive compounds of GTJ, is 9-oxo-10(E),12(E)-ODA, an agonist of PPAR alpha that deserves further investigation. Beyond metabolic improvements, GTJ also exhibits strong antioxidant properties, impacting systemic redox balance and oxidative stress in the liver. This suggests that GTJ’s influence on both metabolic and oxidative pathways could be mediated by PPAR-α activation, coupled with the synergistic action of its diverse bioactive compounds. Future research is warranted to explore these pathways further, particularly the role of GTJ and the broader effects of its phytonutrient complex on MetS, with the aim of proposing a novel functional food.

## Figures and Tables

**Table 1:** Proximate analysis equation according to Official Methods of Analysis of the AOAC International [23].

Parameter	Equation Applied
% Total dry matter	Weight [(dry sample + dish) − dish] initial weight of sample × 100
% Total moisture	100 − % total dry matter
% Protein	% nitrogen × protein factor (6,25)
% Crude Fat	weight [(cup + fat residue) − cup empty] initial sample weight × 100
% Carbohydrate	100 − [(% total moisture + % ash + % protein + % crude fat + % total dietary fiber)]

**Table 2:** (**A**,**B**) Nutritional and chemical composition of GTJ compared with GT. (**C**) Micronutrient composition: the last columns show the recommended daily intake of micronutrients for the Italian adult population (age > 18 years) in good health, moderately active, and daily consumption of 2000 Kcal and the maximum tolerable intake level (UL) according to the WHO and EFSA, on which the recommended daily allowance (RDA) was calculated in %/100 g. * UL values for sodium and potassium represent the nutritional goals for prevention (Suggested Dietary Target, STD). (n.a.) for data that are not available. (*) for *p* < 0.05, (**) for *p* < 0.01, (***) for *p* < 0.001, (****) for *p* < 0.0001 and (n.s.) not significant.

(**A**)							
***Components***		***GT***			***GTJ***	***Statistical Values***	
Water (g/100 g)		91.5 ± 0.3			94 ± 0.6 **	*p* = 0.003 t = 6.455, df = 4	
Proteins (g/100 g)		5.44 ± 0.4			1.31 **** ± 0.06	*p* < 0.0001 t = 17.6856; df = 4	
Lipids (g/100 g)		0.2 ± 0.02			0.19 ± 0.05	n.s.	
Carbohydrates (g/100 g)		4.80 ± 0.49			4.08 ±0.56	n.s.	
Energy (Kcal)		43 ± 2.0			23 ± 3.5 **	*p* = 0.001 t = 8.593, df = 4	
(**B**)							
***Parameters***		***GT***			***GTJ***	***Statistical Values***	
Dry matter (g/Kg^−1^)		85 ± 1.4			158 ± 20.5 **	*p* = 0.0035 t = 6.153; df = 4	
pH		4.4 ± 0.1			4.2 ± 0.1	n.s.	
Brix, °Bx		5.3 ± 0.2			6.6 ± 0.05 ***	*p* = 0.0003 t = 10.92; df = 4	
Titratable acidity (mg%)		0.61 ± 0.01			0.49 ± 0.03 **	*p* = 0.0028 t = 6.573; df = 4	
Total Polyphenols (mg/100 g)		87.6 ± 13.5			82.5 ± 12.7	n.s.	
(**C**)							
***Micronutrients***	***GT***	***GTJ***	***RDA* (mg/die) **		***UL* (mg/die) **	***Statistical Values***	
*Na* (mg/100 g)	90.9 ± 8.1	14.4 ± 4.7 ***	n.a.		2000 *	*p* = 0.0001 t = 14.15, df = 4	
*K* (mg/100 g)	930.1 ± 7.5	113 ± 6.7 ****	n.a.		n.a.	*p* < 0.0001 t = 140.7, df = 4	
*Mg* (mg/100 g)	164.5 ± 10.5	10.8 ± 2.4 ****	170		250	*p* < 0.0001 t = 24.72, df = 4	
*Ca* (mg/100 g)	277.4 ± 9.3	8.8 ± 2.3 ****	1000		2500	*p* < 0.0001 t = 48.56, df = 4	
*Zn* (mg/100 g)	6.7 ± 1.5	0.2 ± 0.03 **				*p* = 0.0017 t = 7.504, df = 4	
*Fe* (mg/100 g)	37.9 ± 5.7	0.84 ± 0.02 ***	Woman	Man	n.a.	*p* = 0.0004 t = 11.26, df = 4	
			18–10	10			
*Cu*	4 ± 0.5	0.04 ± 0.02 ***	0.9		5	*p* = 0.0002 t = 13.71, df = 4	
*Ni*	0.24 ± 0.06	0.007 ± 0.001 **				*p* = 0.0025 t = 6.725, df = 4	
*Mn*	1.7 ± 0.3	0.07 ± 0.04 ***	Woman 2.3	Man 2.7	n.a.	*p* = 0.0007 t = 9.328, df = 4	
*Al*	95.2 ± 4.87	0.65 ± 0.03 ****				*p* < 0.0001 t = 33.63, df = 4	
*Vit. A *(*β-carotene*, mg/100 g)	391 ± 1.3	398 ± 3.6 *	7.5 (μg 1250 RE)		3000 μg	*p* = 0.0339 t = 3.168, df = 4	
*Ascorbic acid, Vit. C* (mg/100 g)	17.04 ± 3.5	4.4 ± 2.4 **	1000		n.a.	*p* = 0.0067 t = 5.159, df = 4	

**Table 3:** Concentrations and retention times of the structural isomers 9-oxo-10(E),12(E)-ODA and 9-oxo-10(E),12(Z)-ODA identified in food matrices, red tomatoes (RT), Golden Tomato (GT), and Golden Tomato Juice (GTJ) by chromatographic analysis. The results are expressed as mean ± standard deviation of two replicates. * indicates statistical difference for *p* < 0.05 of GT vs. RT and vs. GT juice.

Samples	R.T. (Minutes)	9-oxo-10(E),12(E)-ODA (μg/mL)	R.T. (Minutes)	9-oxo-10(E),12(Z)-ODA (μg/mL)
RT	8.1	0.76 ± 0.02	7.4	0.610 ± 0.007
GT	8.1	1.04 ± 0.05 *	7.4	1.43 ± 0.03 *
GTJ	8.2	0.22 ± 0.01	7.5	0.23 ± 0.07

## Data Availability

The data presented in this study are available on reasonable request from the corresponding author.
